# Supplementary material for: Clamping force control of electro–mechanical brakes based on driver intentions
Source: PLoS One. 2020 Sep 24;15(9):e0239608. doi: 10.1371/journal.pone.0239608 (PMC7514108; doi:10.1371/journal.pone.0239608)
Supplement: S1 Appendix — (DOCX) [file pone.0239608.s001.docx]

**S1 Appendix.** Judgement matrices

(1) The judgment matrix *K*_1,_*_dec_* (influence of the accelerator pedal behaviors *C*_1_~*C*_4_ on the driver mild decelerate intention *B*_1_) is as follows:

| $B_{1}$ | $C_{1}$ |  |  | $C_{4}$ |
| --- | --- | --- | --- | --- |
| $C_{1}$ | 1 | 3 | 5 | 7 |
|  | 1/3 | 1 | 3 | 5 |
|  | 1/5 | 1/3 | 1 | 3 |
| $C_{4}$ | 1/7 | 1/5 | 1/3 | 1 |

(2) The judgment matrix *K*_2,_*_dec_* (influence of the accelerator pedal behaviors *C*_1_~*C*_4_ on the driver moderate decelerate intention *B*_2_) is as follows:

| $B_{2}$ | $C_{1}$ |  |  | $C_{4}$ |
| --- | --- | --- | --- | --- |
| $C_{1}$ | 1 | 1/3 | 1/7 | 1/3 |
|  | 3 | 1 | 1/5 | 1/3 |
|  | 7 | 5 | 1 | 5 |
| $C_{4}$ | 3 | 3 | 1/5 | 1 |

(3) The judgment matrix *K*_3,_*_dec_* (influence of the accelerator pedal behaviors *C*_1_~*C*_4_ on the driver strong decelerate intention *B*_3_) is as follows:

| $B_{3}$ | $C_{1}$ |  |  | $C_{4}$ |
| --- | --- | --- | --- | --- |
| $C_{1}$ | 1 | 1/3 | 1/5 | 1/7 |
|  | 3 | 1 | 1/2 | 1/5 |
|  | 5 | 2 | 1 | 1/3 |
| $C_{4}$ | 7 | 5 | 3 | 1 |

(4) The judgment matrix *K*_1,_*_brk_* (influence of the brake pedal behaviors *C*_1_~*C*_4_ on the driver mild brake intention *B*_1_) is as follows:

| $B_{1}$ | $C_{1}$ |  |  | $C_{4}$ | $C_{5}$ | $C_{6}$ | $C_{7}$ |
| --- | --- | --- | --- | --- | --- | --- | --- |
| $C_{1}$ | 1 | 5 | 7 | 3 | 3 | 2 | 1 |
|  | 1/5 | 1 | 5 | 2 | 1/3 | 1/4 | 1/5 |
|  | 1/7 | 1/5 | 1 | 1/5 | 1/5 | 1/5 | 1/7 |
| $C_{4}$ | 1/3 | 1/2 | 5 | 1 | 1/2 | 1/3 | 1/5 |
| $C_{5}$ | 1/3 | 3 | 5 | 2 | 1 | 1/3 | 1/5 |
| $C_{6}$ | 1/2 | 4 | 5 | 3 | 3 | 1 | 1/5 |
| $C_{7}$ | 1 | 5 | 7 | 5 | 5 | 5 | 1 |

(5) The judgment matrix *K*_2,_*_brk_* (influence of the brake pedal behaviors *C*_1_~*C*_7_ on the driver moderate brake intention *B*_2_) is as follows:

| $B_{2}$ | $C_{1}$ |  |  | $C_{4}$ | $C_{5}$ | $C_{6}$ | $C_{7}$ |
| --- | --- | --- | --- | --- | --- | --- | --- |
| $C_{1}$ | 1 | 1/5 | 1/3 | 1/2 | 1/3 | 1/4 | 5 |
|  | 5 | 1 | 5 | 2 | 2 | 1 | 3 |
|  | 3 | 1/5 | 1 | 1/3 | 1/5 | 1/5 | 2 |
| $C_{4}$ | 2 | 1/2 | 3 | 1 | 1/2 | 1/2 | 5 |
| $C_{5}$ | 3 | 1/2 | 5 | 2 | 1 | 1/3 | 3 |
| $C_{6}$ | 4 | 1 | 5 | 2 | 3 | 1 | 7 |
| $C_{7}$ | 1/5 | 1/3 | 1/2 | 1/5 | 1/3 | 1/7 | 1 |

(6) The judgment matrix *K*_3,_*_brk_* (influence of the brake pedal behaviors *C*_1_~*C*_7_ on the driver strong brake intention *B*_3_) is as follows:

| $B_{3}$ | $C_{1}$ |  |  | $C_{4}$ | $C_{5}$ | $C_{6}$ | $C_{7}$ |
| --- | --- | --- | --- | --- | --- | --- | --- |
| $C_{1}$ | 1 | 1/3 | 1/7 | 1/3 | 1/3 | 1/2 | 1 |
|  | 3 | 1 | 1/3 | 2 | 1/3 | 2 | 4 |
|  | 7 | 3 | 1 | 2 | 2 | 3 | 5 |
| $C_{4}$ | 3 | 1/2 | 1/2 | 1 | 2 | 3 | 3 |
| $C_{5}$ | 3 | 3 | 1/2 | 1/2 | 1 | 3 | 3 |
| $C_{6}$ | 2 | 1/2 | 1/3 | 1/3 | 1/3 | 1 | 2 |
| $C_{7}$ | 1 | 1/4 | 1/5 | 1/3 | 1/3 | 1/2 | 1 |
